# Identification of Invasive Aspergillosis in Electronic Health Records

**DOI:** 10.1093/ofid/ofag478

**Published:** 2026-08-08

**Authors:** Emily Rayens, Jessica Skela, Bradley K Ackerson, Magdalena E Pomichowski, Lance B Price, Sara Y Tartof

**Affiliations:** Department of Research & Evaluation, Kaiser Permanente Southern California, Pasadena, California, USA; Department of Research & Evaluation, Kaiser Permanente Southern California, Pasadena, California, USA; Department of Research & Evaluation, Kaiser Permanente Southern California, Pasadena, California, USA; Department of Research & Evaluation, Kaiser Permanente Southern California, Pasadena, California, USA; Department of Environmental and Occupational Health, George Washington University, Washington, DC, USA; Department of Research & Evaluation, Kaiser Permanente Southern California, Pasadena, California, USA; Department of Health Systems Science, Kaiser Permanente Bernard J. Tyson School of Medicine, Pasadena, California, USA

**Keywords:** *Aspergillus*, electronic health records, invasive aspergillosis

## Abstract

**Background:**

Consensus definitions for clinical identification of invasive aspergillosis (IA) are lacking, likely owing to difficulties in diagnosis. While the European Organisation for Research and Treatment of Cancer–Mycoses Study Group (EORTC/MSG) has provided benchmark guidelines to define IA, the criteria are highly restrictive. In this study, we constructed alternative definitions of IA using structured data from electronic health records (EHRs).

**Methods:**

From October 1, 2015, to March 31, 2022, we identified patients with IA from EHRs at Kaiser Permanente Southern California using definitions comprised of combinations of diagnosis codes, antifungal prescriptions, and/or positive mycological findings. Here, we report patient characteristics, including demographic information, underlying comorbidities, and immunosuppression, diagnostic measures, and morbidity and mortality after diagnosis for the population comprising each IA definition.

**Results:**

Between 86 and 4580 individuals were identified as possible IA cases across 7 definitions. All IA definitions that except for that only required positive mycology had similar patient characteristics and outcomes to those meeting 2008 EORTC/MSG criteria. The largest of these cohorts was comprised of 1067 patients, compared with the 86 that met EORTC/MSG criteria. Across IA definitions, all-cause mortality ranged from 11.7% to 33.1% within 6 months following diagnosis. The number of IA cases began trending upward between 2019 and 2020 and continued steadily through the coronavirus disease 2019 pandemic.

**Conclusions:**

IA definitions based on structured data can increase efficiency and identify clinically significant IA cases that might otherwise go unrepresented. Variability in the number of patients identified between definitions further highlights critical deficits in current IA diagnostics.

Invasive aspergillosis (IA) affects roughly 15 000 people in the United States every year, with millions more at risk [1, 2]. Most of these infections are diagnosed in people with compromised immune function, including recipients of solid organ and stem cell transplantation [3], those being treated for cancer [4], and people on immunomodulators for autoimmune and inflammatory diseases [5, 6]. IA is disproportionately represented in immunocompromised patients, with significant morbidity and mortality related to IA. Mortality has been reported to be between 25% and 90%, despite the standard of care in diagnosing and treating infections [2, 7–9]. The number of mortality reports with IA listed as a cause of death has steadily risen since 2013, alongside reports of immunocompromising conditions [10]. These numbers likely underestimate the true burden of disease due to widespread underdiagnosis and underreporting of invasive fungal infections [11, 12].

Diagnosing IA with certainty can be difficult as it has been estimated that up to 75% of patients with IA may have negative cultures [13], and a lack of laboratory confirmation can delay diagnosis and limit susceptibility testing in the lab. Conversely, a positive culture can be indicative of subclinical colonization, unrelated to the patient's symptoms, as *Aspergillus* is a ubiquitous organism and most individuals breathe in hundreds of conidia per day [14, 15]. Additionally, *Aspergillus* is associated with a spectrum of disease presentations, with varying recommendations for clinical management, which has led to longstanding debate regarding consistent and reliable identification of IA cases. To this end, the European Organisation for Research and Treatment of Cancer–Mycoses Study Group (EORTC/MSG) developed a standard definition for identifying IA cases with the highest possible confidence. In short, these criteria limit the population to immunocompromised persons, who are most susceptible to invasive disease, those with visualization of *Aspergillus*, and those who have disease that is evident by imaging. These criteria are reasonable for clinical trials and other circumstances that require a high degree of specificity and a high level of homogeneity in IA cases. However, reviewing cases for EORTC/MSG criteria requires extensive effort, with evidence that may be documented over weeks or months. Further, this population does not represent clinically significant patients with IA who are still being managed with antifungal treatments. Therefore, to explore the IA patient populations unrepresented by the EORTC/MSG definition, we created 6 alternative definitions of IA using readily available structured electronic health record (EHR) data and compared population characteristics, temporal trends, and morbidity and mortality by IA definition.

## METHODS

### Study Setting

Kaiser Permanente Southern California (KPSC) is a large, integrated prepaid health care system serving >4.9 million members in Southern California who are representative of a diverse region [16, 17]. KPSC insurance is covered through employer-based plans, individual plans, and Medicare or state-subsidized health care [16]. Patient information is collected in the EHR, including demographic and socioeconomic characteristics, diagnoses, procedures, vaccines, medications, and laboratory testing from all care settings (inpatient, emergency department, outpatient, and virtual encounters). Ethical approval for the study was obtained from the KPSC Institutional Review Board, which waived the requirement for informed consent.

### Study Population

KPSC members aged ≥18 years meeting at least 1 outcome definition between October 1, 2015, and March 31, 2022, with at least 6 months of continuous KPSC membership before the diagnosis date (allowing for a 31-day gap in membership) were eligible for inclusion in the analysis. We excluded individuals with any history of aspergillosis before the study period identified by the International Classification of Diseases, 9th revision (ICD-9), diagnosis code for aspergillosis (117.3).

### Invasive Aspergillosis Definitions

We used 7 definitions for IA, including (1) positive mycology, (2) positive mycology and antifungal prescription, (3) diagnosis code only, (4) code and antifungal prescription, (5) code and positive mycology, (6) code, positive mycology, and antifungal prescription, and (7) cases meeting EORTC/MSG criteria.

The diagnosis date was defined as the date that the patient first met all criteria for the definition. The ICD-10 diagnosis codes used for identification included B44.0 (invasive aspergillosis), B44.7 (disseminated aspergillosis), and B44.9 (aspergillosis, unspecified). Mycological findings, including culture, pathology, and molecular testing, were only considered from samples pulled from respiratory and sterile sites for relevance (Supplementary Methods). Galactomannan testing from bronchoalveolar lavage and blood was considered positive with an optical density ≥0.5 [18]. As clinical management of IA may begin before official diagnosis, antifungal prescriptions (posaconazole, itraconazole, isavuconazole, voriconazole, and amphotericin B) were considered up to 31 days before and 31 days after the diagnosis date (Supplementary Methods). For the EORTC/MSG definition, all patients with a relevant ICD-10 diagnosis code were considered [18]. Proven IA cases were defined as those with positive culture or histology from a sterile source. Probable IA cases were defined as those who (A) had a positive mycology result (culture, pathology, or galactomannan test) from a respiratory source, (B) were deemed at clinical risk of IA by diagnosis of relevant immunosuppressing condition, medication prescription, or laboratory result indicating persistent neutropenia (Supplementary Methods, Supplementary Tables 1–2), and (C) had relevant imaging ordered, including computed tomography (CT) scan or x-ray with findings supportive of IA diagnosis, including lesions and air crescent signs. The final set of cases meeting proven or probable criteria were reviewed by an infectious disease physician (B.K.A.) to confirm sufficient evidence for IA diagnosis using the 2008 EORTC/MSG guidelines.

### Analysis

Demographic and diagnostic information (Supplementary Methods), as well as morbidity (including hospital and intensive care unit [ICU] admission and use of mechanical ventilation) and mortality (at 180 and 365 days after diagnosis), were described during the study period for patients in all IA cohorts. As the study period included years with <12 months of data, the number of cases per year was divided by the number of months available for that particular year. Similarly, the number of cases per month was divided by the number of years with data for that month. All analyses were conducted using SAS, version 9.4 or later, software (SAS Institute, Cary, NC, USA).

## RESULTS

Between 2015 and 2022, 4580 individuals were positive for *Aspergillus* by mycological testing of samples from sterile and respiratory sites (Table 1). During that same period, 679 individuals had positive mycology and antifungal prescription, 1067 had an IA diagnosis code, 680 had an IA diagnosis code and antifungal prescription, 415 had a diagnosis code and positive mycology, 323 individuals had a diagnosis code, positive mycology, and antifungal prescription, and 86 were confirmed as proven or probable IA cases by 2008 EORTC/MSG criteria.

**Table 1.** Characteristics of Individuals With Invasive Aspergillosis at Kaiser Permanente Southern California, 2015–2022

	(1) Mycology n = 4580	(2) Mycology + Antifungals n = 679	(3) Code n = 1067	(4) Code + Antifungals n = 680	(5) Code + Mycology n = 415	(6) Code + Mycology + Antifungals n = 323	(7) 2008 EORTC/MSG Criteria n = 86
Age	…	…	…	…	…	…	…
Mean (SD), y	63.6 (15.3)	62.7 (14.4)	63.0 (14.8)	63.1 (15.0)	63.7 (14.4)	63.4 (14.8)	57.2 (15.9)
Median (IQR), y	66.0 (55.0–75.0)	65.0 (56.0–73.0)	66.0 (56.0–73.0)	66.0 (56.0–73.0)	66.0 (57.0–74.0)	67.0 (56.0–74.0)	60.0 (46.0–70.0)
Age group, n (%)	…	…	…	…	…	…	…
18–44 y	559 (12.2)	79 (11.6)	129 (12.1)	80 (11.8)	48 (11.6)	38 (11.8)	21 (24.4)
45–64 y	1518 (33.1)	244 (35.9)	357 (33.5)	222 (32.6)	133 (32.0)	99 (30.7)	28 (32.6)
65–79 y	1933 (42.2)	307 (45.2)	480 (45.0)	311 (45.7)	196 (47.2)	159 (49.2)	36 (41.9)
80+ y	570 (12.4)	49 (7.2)	101 (9.5)	67 (9.9)	38 (9.2)	27 (8.4)	1 (1.2)
Code, n (%)	…	…	…	…	…	…	…
B44.0	N/A	N/A	199 (18.7)	151 (22.2)	88 (21.2)	81 (25.1)	45 (52.3)
B44.7	N/A	N/A	14 (1.3)	10 (1.5)	7 (1.7)	7 (2.2)	1 (1.2)
B44.9	N/A	N/A	854 (80.0)	519 (76.3)	320 (77.1)	235 (72.8)	40 (46.5)
Sex, n (%)	…	…	…	…	…	…	…
Female	2346 (51.2)	305 (44.9)	501 (47.0)	313 (46.0)	195 (47.0)	152 (47.1)	40 (46.5)
Male	2234 (48.8)	374 (55.1)	566 (53.0)	367 (54.0)	220 (53.0)	171 (52.9)	46 (53.5)
Race/ethnicity, n (%)	…	…	…	…	…	…	…
Asian	514 (11.2)	79 (11.6)	119 (11.2)	66 (9.7)	49 (11.8)	33 (10.2)	15 (17.4)
Black	369 (8.1)	70 (10.3)	88 (8.2)	59 (8.7)	39 (9.4)	33 (10.2)	10 (11.6)
Hispanic	1296 (28.3)	233 (34.3)	343 (32.1)	219 (32.2)	134 (32.3)	110 (34.1)	33 (38.4)
Other	165 (3.6)	22 (3.2)	22 (2.1)	18 (2.6)	9 (2.2)	8 (2.5)	4 (4.7)
White	2236 (48.8)	275 (40.5)	495 (46.4)	318 (46.8)	184 (44.3)	139 (43.0)	24 (27.9)
EORTC/MSG confirmation, n (%)		…	…	…	…	…	…
Yes	83 (1.8)	68 (10.0)	86 (8.1)	74 (10.9)	76 (18.3)	69 (21.4)	86 (100.0)
Proven	24 (0.5)	23 (3.5)	25 (2.3)	23 (3.4)	21 (5.1)	21 (6.5)	25 (29.1)
Probable	59 (1.3)	45 (6.6)	61 (5.7)	51 (7.5)	55 (13.3)	48 (14.9)	61 (70.9)
Weighted Charlson score, n (%)		…	…	…	…	…	…
0	588 (12.8)	28 (4.1)	47 (4.4)	20 (2.9)	11 (2.7)	5 (1.5)	3 (3.5)
1–2	1409 (30.8)	150 (22.1)	226 (21.2)	139 (20.4)	89 (21.4)	60 (18.6)	14 (16.3)
3–4	929 (20.3)	154 (22.7)	208 (19.5)	139 (20.4)	85 (20.5)	75 (23.2)	18 (20.9)
5+	1654 (36.1)	347 (51.1)	586 (54.9)	382 (56.2)	230 (55.4)	183 (56.7)	51 (59.3)
Immunocompromised, n (%)		…	…	…	…	…	…
Yes	1584 (34.6)	332 (48.9)	483 (45.3)	334 (49.1)	188 (45.3)	155 (48.0)	73 (84.9)
HSCT	63 (1.4)	34 (5.0)	47 (4.4)	34 (5.0)	13 (3.1)	11 (3.4)	9 (10.5)
SOT	170 (3.7)	87 (12.8)	108 (10.1)	67 (9.9)	40 (9.6)	35 (10.8)	24 (27.9)
Immunosuppressive medications	537 (11.7)	227 (33.4)	207 (19.4)	156 (22.9)	83 (20.0)	69 (21.4)	56 (65.1)
Immunosuppressive conditions	767 (16.7)	239 (35.2)	417 (39.1)	285 (41.9)	158 (38.1)	129 (39.9)	56 (65.1)
Neutropenia	71 (1.6)	51 (7.5)	60 (5.62)	47 (6.9)	21 (5.1)	19 (5.9)	17 (19.8)
Mycology attempted, n (%)	…	…	…	…	…	…	…
Yes	4580 (100.0)	679 (100.0)	632 (59.2)	469 (69.0)	415 (100.0)	323 (100.0)	81 (94.2)
Fungal culture	3230 (70.5)	603 (88.8)	490 (45.9)	376 (55.3)	383 (92.3)	302 (93.5)	76 (88.4)
Microscopy^a^	1758 (38.4)	195 (28.7)	122 (11.4)	91 (13.4)	92 (22.2)	73 (22.6)	26 (30.2)
Galactomannan	869 (19.0)	328 (48.3)	368 (34.5)	284 (41.8)	221 (53.3)	178 (55.1)	42 (48.8)
Mycology positive, n (%)	…	…	…	…	…	…	…
Yes	4580 (100.0)	679 (100.0)	415 (61,1)	323 (47.5)	415 (100.0)	323 (100.0)	76 (88.4)
Fungal culture	2835 (61.9)	481 (70.8)	330 (30.9)	261 (38.4)	330 (79.5)	261 (80.8)	66 (76.7)
Microscopy	1498 (32.7)	113 (16.6)	63 (5.9)	46 (6.8)	63 (15.2)	46 (14.2)	20 (22.3)
Galactomannan	440 (9.6)	184 (27.1)	103 (9.7)	87 (12.8)	103 (24.8)	87 (26.9)	13 (15.1)
Imaging ordered,^b^ n (%)	3643 (79.5)	658 (96.9)	1013 (94.9)	666 (97.9)	410 (98.8)	322 (99.7)	86 (100.0)
Antifungal use,^c^ n (%)	…	…	…	…	…	…	…
Before and/or after diagnosis	679 (14.8)	679 (100.0)	680 (64.7)	680 (100.0)	323 (77.8)	323 (100.0)	74 (86.0)
Before diagnosis	156 (3.4)	156 (23.0)	193 (18.1)	193 (28.4)	64 (15.4)	64 (19.8)	26 (30.2)
After diagnosis	637 (13.9)	637 (93.8)	606 (56.8)	606 (89.1)	305 (73.5)	305 (94.4)	68 (79.1)

Abbreviations: CT, computerized tomography scan; EORTC/MSG, European Organisation for Research and Treatment of Cancer–Mycoses Study Group; HSCT, hematopoietic stem cell transplant; IQR, interquartile range; n, number; N/A, not applicable; SOT, solid organ transplant; y, year.

^a^Results include samples where any fungal elements were noted in reporting.

^b^Orders placed for CT scan or x-ray 31 days before or after the diagnosis date related to chest, thorax, head, brain, spine, or skull.

^c^Azole and/or amphotericin B prescribed in the 31 days before and/or after diagnosis date, as described.

Across the 7 IA definitions, the mean age (SD) ranged from 57.1 (16.0) to 63.7 (14.4) years (Table 1). Aside from Definition 1, each definition identified more male patients than female. In most definitions, the cohort was largely identified as non-Hispanic White (40.5%–48.8%), followed by Hispanic (28.3%–34.4%). However, in the EORTC/MSG-confirmed cohort, the largest racial or ethnic group was Hispanic (38.4%), with lower frequency of non-Hispanic White (27.9%) individuals and greater frequency of Asian individuals (17.4%) observed compared with the other definitions.

For definitions that required a diagnosis code (Definitions 3, 4, 5, and 6), the code for unspecified aspergillosis was most frequently used (72.8%–80.0%). The code for unspecified aspergillosis was also used more frequently than B44.0 (invasive aspergillosis) in the EORTC/MSG-confirmed cohort (61.6%). Most EORTC/MSG-confirmed cases met probable IA criteria (70.9%). Of the 86 EORTC/MSG-confirmed cases during this period, at least 69 (80.2%) were picked up by other definitions, although they represented only 1.8%–21.4% of cases in those definitions. Definition 3 (diagnosis code only) was the only alternative definition to pick up 100% of EORTC/MSG-confirmed cases.

Most definitions identified patients with a Charlson Comorbidity score of 5 or more (54.9%–66.3%), apart from Definition 1 (35.1%). Similarly, roughly half of patients with a diagnosis code were immunocompromised at diagnosis date (45.3%–49.1%). In comparison, 79.1% of individuals who met EORTC/MSG criteria were considered immunocompromised. The remaining 20.9% of the EORTC/MSG cohort met proven criteria, which only require positive mycology from a sterile site. Four of the 7 definitions required positive mycology; however, in Definitions 3 and 4, 59.2%–69.0% had relevant mycological testing conducted and 38.9%–47.5% returned positive results. In the EORTC/MSG cohort, 94.2% had mycological testing performed within the ±31-day window, with 88.4% returning a positive result. Antifungals were prescribed in the majority of patients (63.7%–100%), except those with positive mycology only (14.8%). Antifungal prescriptions were more frequently observed after diagnosis (56.8%–94.4%) vs before diagnosis (15.4%–30.2%).

All-cause morbidity and mortality were substantial across all definitions. In the 30 days before and after IA diagnosis, between 24.7% and 75.6% of patients were hospitalized, 9.8%–30.5% were admitted to the ICU, and 9.5%–29.3% received mechanical ventilation (Table 2). For hospital and ICU admission, the highest frequencies were observed in those captured in Definition 2, and the lowest in those identified by Definition 1. All-cause mortality generally ranged from 26.7% to 33.1% and 33.3% to 38.1% in the 6 months and 1 year following the diagnosis date, respectively. In the cohort captured in Definition 1, lower mortality was observed (11.7% and 14.9%, respectively) (Table 2).

**Table 2.** Morbidity and Mortality Associated With Invasive Aspergillosis at Kaiser Permanente Southern California, 2015–2022

	(1) Mycology n = 4580	(2) Mycology + Antifungals n = 679	(3) Code n = 1067	(4) Code + Antifungals n = 680	(5) Code + Mycology n = 415	(6) Code + Mycology + Antifungals n = 323	(7) 2008 EORTC/MSG Criteria n = 86
Hospital admission,^a^ n (%)	1133 (24.7)	410 (61.8)	637 (59.7)	399 (58.7)	234 (56.4)	195 (60.4)	65 (75.6)
ICU admission,^a^ n (%)	447 (9.8)	207 (31.2)	228 (21.4)	156 (22.9)	93 (22.4)	79 (24.5)	24 (27.9)
Mechanical ventilation,^a^ n (%)	435 (9.5)	199 (30.0)	221 (20.7)	151 (22.2)	97 (23.4)	81 (25.1)	20 (23.3)
All-cause mortality, n (%)	…	…	…	…	…	…	…
180 d	537 (11.7)	225 (33.9)	323 (30.3)	197 (29.0)	119 (28.7)	97 (30.0)	27 (31.4)
365 d	683 (14.9)	357 (38.8)	360 (33.7)	227 (33.4)	138 (33.3)	116 (35.9)	31 (36.0)

Abbreviations: d, days; EORTC/MSG, European Organisation for Research and Treatment of Cancer–Mycoses Study Group; ICU, intensive care unit; n, number.

^a^Occurring in the 30 days before or after the diagnosis date.

**Figure 1.**
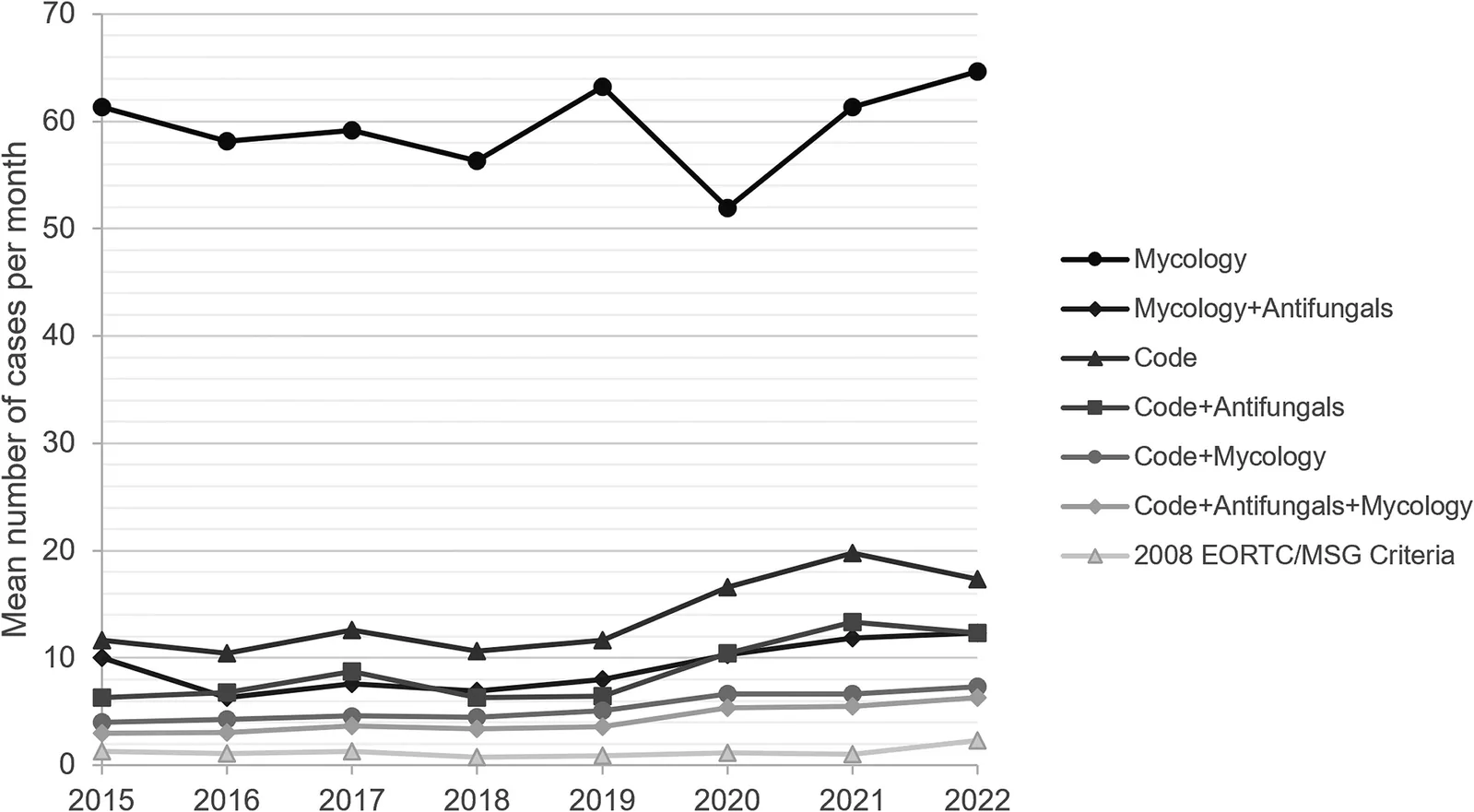
Invasive aspergillosis cases per year at Kaiser Permanente Southern California, 2015–2022. Number of patients meeting each invasive aspergillosis definition, divided by the number of months for which data were available in that year. Abbreviation: EORTC-MSG, European Organisation for Research and Treatment of Cancer–Mycoses Study Group.

**Figure 2.**
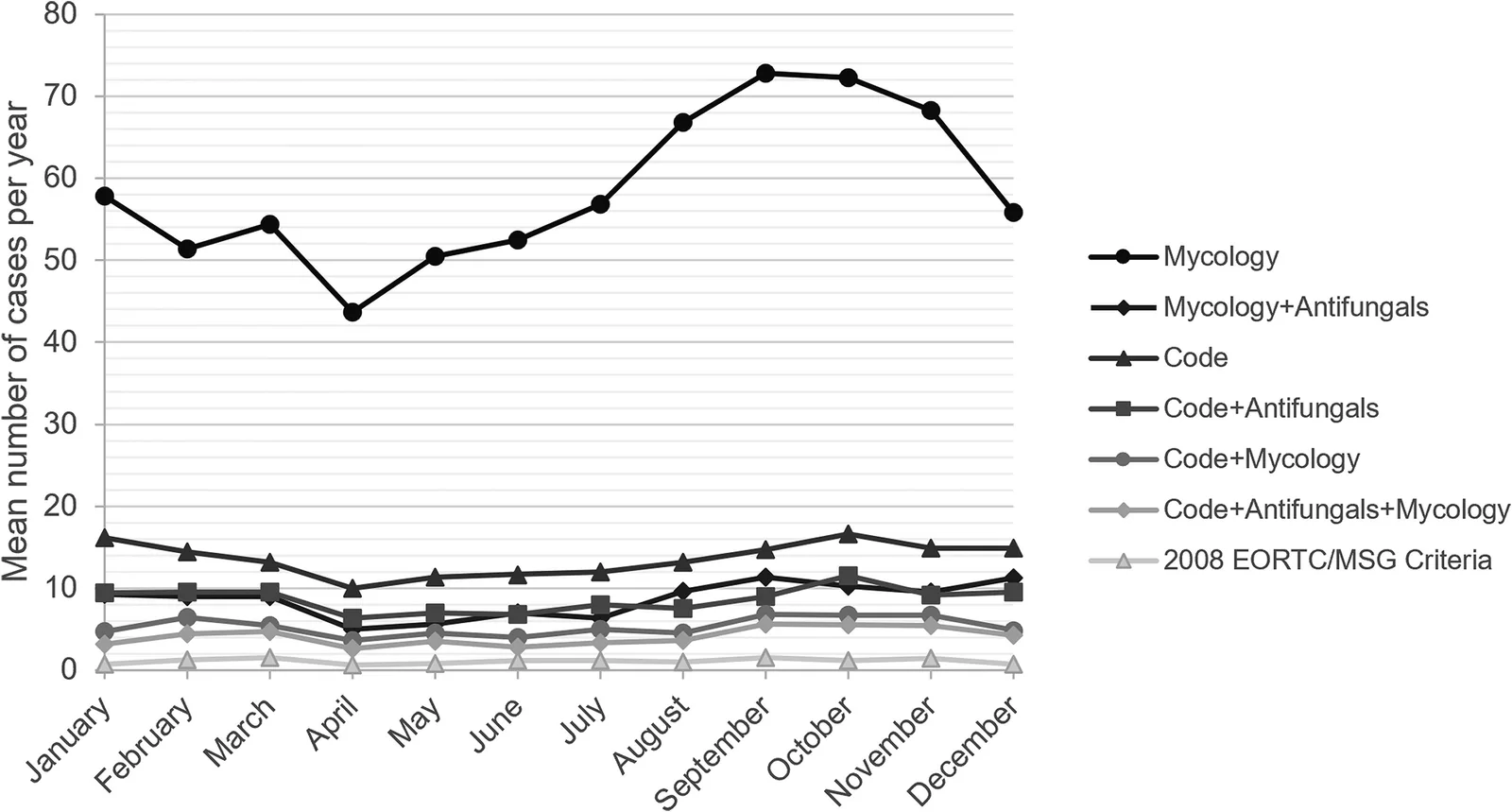
Invasive aspergillosis cases per month at Kaiser Permanente Southern California, 2015–2022. Number of patients meeting each invasive aspergillosis definition, divided by the number of years for which data were available in that month. Abbreviation: EORTC-MSG, European Organisation for Research and Treatment of Cancer–Mycoses Study Group.

By most definitions, the number of IA cases began trending upward between 2018 and 2019 (Figure 1). The exception to this was Definition 1, for which there was a significant drop in 2020 before resuming prepandemic numbers in 2021, and the EORTC/MSG-confirmed cases, for which there was not a trend in the number of cases during the study period. By most definitions, the number of IA cases was lowest between the months of April and June, and seasonality was observed in Definition 1, peaking in September (Figure 2).

## DISCUSSION

We report population characteristics, temporal trends, and morbidity and mortality for 7 definitions of IA, including those identified by the internationally accepted EORTC/MSG guidelines. We found that multiple IA definitions identified a much larger patient cohort that shared patient characteristics and burden of morbidity and mortality similar to those of the EORTC/MSG population, indicating that the EORTC/MSG definition likely underrepresents patients with IA and that alternative definitions may be useful in a variety of settings.

Identifying IA cases with confidence is complicated due to nonspecific symptoms and diagnostic testing with low or variable sensitivity. It is possible that a coded diagnosis may not translate to relevant clinical disease, especially when the type of aspergillosis is unspecified and there are no associated relevant mycological findings. To address this limitation, the EORTC/MSG developed a standard definition for identifying IA cases [18]. This approach identifies cases with high confidence but may underrepresent IA cases that are still being managed clinically [19]. In the alternative definitions that included positive identification of *Aspergillus* and/or evidence of IA treatment with azoles or amphotericin B, patient demographics were similar to those of the EORTC/MSG-confirmed cohort. It is also of note that more specific ICD-10 codes for IA fail to capture all IA patients as 61.6% of the EORTC/MSG-confirmed cohort had been diagnosed with unspecified aspergillosis but met all diagnostic criteria for IA.

Although we observed similarities in the patient characteristics of the alternative IA definitions, there are also several points of interest. In patients identified by code, only 60% had mycological testing performed within ±31 days of diagnosis. Indications for code may have come from outside testing not captured in EHRs or even changes noted in imaging results as 95% of the patients in this cohort had relevant imaging orders in the same period. However, only around 64% were prescribed antifungal treatment in the days surrounding diagnosis. Definitions that require mycological findings and treatment may be a better representation of empirical treatment of IA, while still appreciating a more comprehensive patient population.

The frequencies of morbidity outcomes, defined as admission to the hospital or ICU and use of mechanical ventilation, were similar across patient cohorts requiring a diagnosis code. However, the hospital and ICU admission frequencies in definitions requiring a diagnosis code were lower than those observed in the EORTC/MSG-confirmed cohort (75.6% vs 56.4%–60.4% and 27.9% vs 21.4%–24.5%, respectively), although they remained substantial. It is important to note that all-cause mortality was similar between IA cohorts requiring a diagnosis code and EORTC/MSG-confirmed at 28.7%–30.3% vs 31.4% and 33.3%–33.7% vs 36.0% at 6 months and 1 year from diagnosis, respectively. Overall, the cohorts meeting alternative IA definitions requiring a diagnosis code were similar to that meeting EORTC/MSG criteria, with 4–10 times as many patients.

The frequency of IA did not appear to be impacted by the coronavirus disease 2019 (COVID-19) pandemic. Although the number of patients with positive *Aspergillus* cultures dropped in 2020, other IA definitions were not similarly affected when coded diagnosis and antifungal prescriptions were considered. COVID-19-associated pulmonary aspergillosis (CAPA) has been considered as a serious potential complication of COVID-19 in critically ill patients [20, 21]; however, there was not a notable impact on the number of diagnosed patients in this setting. In addition to literature describing the impact of COVID-19 and other impacts on trends in burden of *Aspergillus* over time, increasing attention has been devoted to the seasonality of *Aspergillus* and associated disease [22–24]. The identification of airborne *Aspergillus* has been shown to peak in the summer or fall after periods of low precipitation and high temperatures [22–24]. This is supported in the current study, where the number of patients with a positive *Aspergillus* culture peaked in September. However, this observed seasonality did not appear to transfer to diagnosed IA cases. The literature on this topic is limited, but a study of hematopoietic stem cell transplant patients in diverse climates (Seattle, WA, USA, and Houston, TX, USA) found evidence of seasonality associated with IA in Seattle but not Houston, where total spore counts were lower and not variable by climate [24].

This study had several strengths. First, the size and diversity of the study cohort enhanced the generalizability of our findings. Second, KPSC's comprehensive EHR system enabled broad capture of aspergillosis diagnoses, including mycological testing and antifungal prescription across all settings of care, as well as demographic and clinical covariates and mortality in the year following the diagnosis date. This study also had potential limitations. Misclassification and missing data were possible if mycological testing or antifungal prescriptions were received outside of KPSC; however, all individuals in this study were required to have at least 6 months of KPSC membership before diagnosis, and outside care is captured in the EHR through reimbursement for claims. Additionally, our cohort meeting EORTC/MSG criteria for diagnosis was reviewed based on the 2008 criteria. The diagnostic guidelines have since been updated [25], with limited additions to the immunosuppressive medication list and inclusion of *Aspergillus* polymerase chain reaction (PCR) results for mycological evidence. However, we expect that the definition used in this study identifies the vast majority of patients who would meet the updated EORTC/MSG criteria, as the additions to the immunosuppressive medication lists were minor. Additionally, PCR testing was not used at KPSC during the study period, so additional patients could not have been identified by PCR [26, 27]. Finally, the number of patients who met EORTC/MSG criteria was small, which may reflect the limitations of retrospective identification of IA and incomplete capture of patient data due to testing or care that occurred outside of KPSC.

In conclusion, we present several alternative definitions for the identification of IA cohorts using structured data from the EHR that share similar patient characteristics, temporal trends, and burden of morbidity and mortality as those of the EORTC/MSG population. While variability in the number of patients identified between definitions highlights critical deficits in current IA diagnostics, these definitions may allow for the capture and analysis of clinically significant IA cases that might otherwise go unrepresented.

## Supplementary Material

ofag478_Supplementary_Data

## Supplementary Data

Supplementary materials are available at Open Forum Infectious Diseases online. Consisting of data provided by the authors to benefit the reader, the posted materials are not copyedited and are the sole responsibility of the authors, so questions or comments should be addressed to the corresponding author.
