# Supplementary material for: Identification of Invasive Aspergillosis in Electronic Health Records
Source: Open Forum Infect Dis. 2026 Aug 8;13(8):ofag478. doi: 10.1093/ofid/ofag478 (PMC13507318; doi:10.1093/ofid/ofag478)
Supplement: Supplementary file 1 [file ofag478_supplementary_data.docx]

**Supplementary Materials**

**Table of Contents**

Supplementary Methods

Supplementary Table 1. Immunocompromised Condition Codes.

Supplementary Table 2. Immunosuppressing Medications.

**Supplementary Methods**

To meet the 2008 EORTC/MSG criteria for proven invasive aspergillosis (IA), positive culture or pathology had to come from a sterile source. Probable IA cases were identified in patients that (A) had a positive mycology result (culture, pathology, or Galactomannan) from a respiratory source, (B) had diagnosis of relevant immunosuppressing condition, medication prescription, or laboratory result indicating persistent neutropenia, and (C) had relevant imaging ordered, including computed tomography (CT) scan and x-ray. The specifics of these criteria are as follows:

Mycology

- Sample sources
  - Sterile: biopsy, brain, pleural fluid, peritoneal fluid, joint fluid, bone, tissue, fine needle aspirate, transbronchial aspirate, ascites fluid
  - Respiratory: bronchoalveolar lavage (BAL), sputum, bronchial brushings, bronchial washings, sinus, transtracheal aspirate
  - Galactomannan only: blood
- Indication of positive results
  - Fungal culture: positive identification of *Aspergillus* species in results field and/or notes
  - Pathology: Positive for *Aspergillus* or fungal species not otherwise specified
    - Example: “fungal organisms found”; “abundant fungal hyphae”
  - Galactomannan testing (CPT code 87305): optical density ≥0.5.

Immunosuppression

- Immunocompromised conditions diagnosed at any time prior to diagnosis date with ICD-9 or ICD-10 code (**Supplementary Table 1**).
- Immunosuppressive medications collected from inpatient, outpatient, and claims pharmacy data (**Supplementary Table 2**).
  - Prolonged use of corticosteroids at a mean minimum dose of 0.3 mg/kg/day of prednisone equivalent for >3 weeks prior to diagnosis.
  - Treatment with recognized T-cell immunosuppressants, such as cyclosporine, tacrolimus, monoclonal antibodies, or nucleoside analogues in the 90 days prior to diagnosis.

Imaging

- CT or x-ray orders of the chest, thorax, head, brain, spine, skull were considered for review.

Additional Variables evaluated at the following timepoints

- Up to 1 year prior to diagnosis date:
  - Charlson comorbidity index
- At diagnosis date:
  - Demographic and socioeconomic variables: age, sex, race/ethnicity
- In the 6 months prior to and following diagnosis date:
  - Antifungals: posaconazole, itraconazole, isavuconazole, voriconazole, amphotericin B; collected from inpatient, outpatient, and claims pharmacy data
  - Mycology from sterile and respiratory sources, as described above
  - Imaging, as described above
- In the 30 days prior to and following diagnosis date:
  - Hospital admission
  - Intensive care unit (ICU) admission
  - Mechanical ventilation, identified by procedure codes

**Supplementary Table 1. Immunocompromised Condition Codes.**

|  | **ICD-9 Code** | **ICD-10 Code** |
| --- | --- | --- |
| Hematopoietic stem cell transplant | N/A | Z94.84, Z94.81, V42.82, V42.81 |
| Solid organ transplant | N/A | Z94.0, Z94.1, Z94.2, Z94.3, Z94.4, Z94.82, Z94.83, Z94.89, Z94.9, V42.0, V42.1, V42.6, V42.7, V42.84, V42.83, V42.89, V42.9 |
| Leukemia/lymphoma | 201*, 200.30, 200.80, 202.30, 202.50, 202.6*, 202.80, 202.81, 202.83, 202.87, 202.92, 203.80, 202.9*, 203.81, 238.79, 273.3, 238.4, 238.72, 238.73, 238.74, 238.75, 203*, 204*, 205*, 206*, 207*, 208*, 277.89 | C81*- C86*, C88.*, C90.*- C96.*, D45, D46.* |
| Congenital immunodeficiencies | 284.09, 284.89, 284.2, 284.9, 288.01, 288.1, 277.6, 277.2, 279*, 258.1, 270.2, 334.8, 757.31, 759.0, 759.1, 759.2, 759.3, 759.4, 759.7, 759.89, 759.9 | D61.09, D61.3, D61.82, D61.9, D70.0, D71, D80.0, D80.1, D80.5, D80.8, D81.*, D82.*, D83.*, D84.0, D84.1, D84.89, D84.9, D89.81*, D89.82, D89.9, E31.0, E70.330, G11.3, Q82.4, Q89.0* |
| Asplenia | 282.60, 282.61, 282.62, 282.63, 282.64,282.41, 282.42, 282.68, 282.69, 289.59, 759.0 | D57.00, D57.01, D57.02, D57.1, D57.20, D57.21, D57.211, D57.212, D57.219, D57.40, D57.411, D57.412, D57.419, D57.80, D57.81, D57.811, D57.812, D57.819, D73.0, Q89.01, Q89.09, V45.79, Z90.81, |
| Chronic granulomatous disease | 288.1 | D71 |
| Severe combined immunodeficiencies | 279.2 | D81.1 |

*All subcodes found below the one listed were included in the definition.

ICD, International Classification of Disease; N/A , not applicable

**Supplementary Table 2. Immunosuppressing Medications.**

| **Medication Category** | **Medication** |
| --- | --- |
| Corticosteroids | Beclomethasone, Betamethasone, Budesonide, Cortisone, Deflazacort, Dexamethasone, Methylprednisolone, Prednisolone, Prednisone, Triamcinolone |
| T-cell Immunosuppressants | Midostaurin, Bafecitinib, Imatinib, Nilotinib, Desatinib, Bosutinib, Ponatinib, Venetoclax, Tacrolimus, Sirolimus, Timsirolimus, Cyclosporine, Mycophenolate Mofetil, Leflunomide, Tofacitinib, Baricitinib, Ruxolitinib, Oclacitinib, Upadacitinib, Fedratinib, Abrocitinib, Pacritinib, Deucravacitinib, Ritlecitinib, Momelotinib, Abatacept, Belatacept, Anakinra, Canakinumab, Riolnacept, Tocilizumab, Sarilumab, Siltixumab |
| Nucleoside analogues | Capecitabine, Cladribine, Clofarabine, Cytarabine, Fludarabine Phos, Floxuridine, Fluorouracil, Gemcitabine, Hydroxyurea, Mercaptopurine, Pentostatin, Thioguanine |
